# N- and C-terminal regions of the small heat shock protein IbpA from *Acholeplasma laidlawii* competitively govern its oligomerization pattern and chaperone-like activity†

**DOI:** 10.1039/c9ra10172a

**Published:** 2020-02-26

**Authors:** Liliya S. Chernova, Mikhail I. Bogachev, Vitaly V. Chasov, Innokentii E. Vishnyakov, Airat R. Kayumov

**Affiliations:** Kazan Federal University 18 Kremlevskaya street 420008 Kazan Russia kairatr@yandex.ru +7-843-233-78-02; Institute of Cytology, Russian Academy of Sciences 4 Tikhoretsky Avenue 194064 St-Petersburg Russia innvish@gmail.com +7-812-297-03-28; St. Petersburg Electrotechnical University 5 Professor Popov street 197376 St. Petersburg Russia; Peter the Great St.Petersburg Polytechnic University 29 Polytechnicheskaya street 195251 St-Petersburg Russia

## Abstract

Small heat shock proteins (sHSPs) are ubiquitous molecular chaperones preventing the irreversible denaturation of proteins. While in *Escherichia coli* two sHSPs IbpA and IbpB work in strong cooperation, the sole Mollicute with free-living ability *Acholeplasma laidlawii* carries a single gene encoding the sHSP protein *Al*IbpA. *In vitro*, independently of the temperature, *Al*IbpA forms a heterogeneous mixture of approximately 24-mer globules, fibrils and huge protein aggregates. The removal of either 12 or 25 N-terminal amino acids led to the formation of fibrils and enhanced the protein ability to prevent the temperature-induced aggregation of insulin, assuming the fibrillar form as an active protein. In turn, the deletion of the C-terminus or substitution of C-terminal LEL motif by SEP decreased the temperature stability of *Al*IbpA and eliminated its chaperone function completely, although the protein remained predominantly in a globular state. This suggests that the C-terminal LEL motif is necessary for the chaperon-like activity of *Al*IbpA and fibril formation. Double N- and C-terminal truncations abolished both the chaperone-like activity and huge oligomer formation. Since the globular form of sHSPs is considered as their inactive form, our data suggest that the N-terminus of *Al*IbpA is responsible for the huge globule (low-active form) formation and behaves as an intramolecular inhibitor of the fibrils (active form) formation and substrates binding. Taken together these data demonstrate non-trivial properties of *Al*IbpA, in which the competitive action of N- and C-termini governs the equilibrium between either fibrillar or globular structures representing a possible molecular mechanism of the *Al*IbpA activity regulation.

## Abstract 2

The CTD provides fibrils (active form) formation. The NTD leads to globules formation and behaves as an intramolecular inhibitor of CTD. Their competition governs the equilibrium between either fibrills or globules regulating the *Al*IbpA activity.

## Graphical abstract

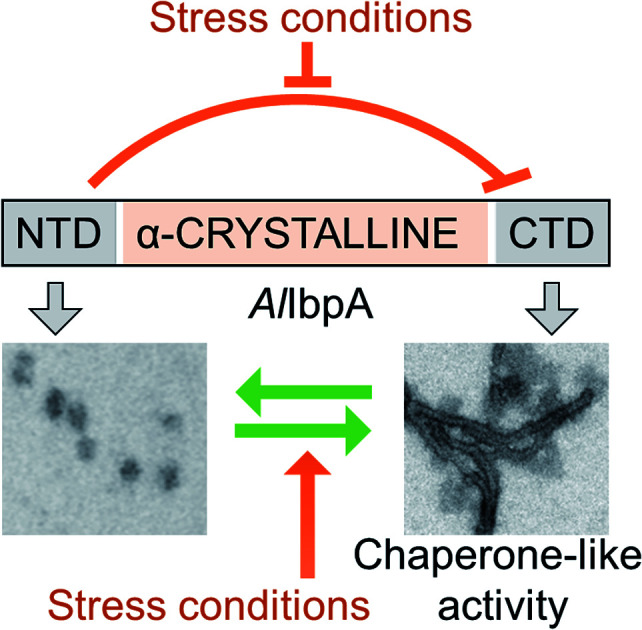

## Introduction

Protein aggregation induced by heat-shock, and oxidative or salt stress is a major threat for cell viability. Small heat shock proteins (sHSPs) represent an abundant and ubiquitous family of molecular chaperones that are believed to prevent irreversible aggregation of other cellular proteins under stress conditions.^1–4^

In cells sHSPs form huge homo- and/or heterooligomeric complexes consisting of typically between 9 and 48 subunits.^1,5,6^ This assembly of sHSPs involves several parts of the subunits in a flexible manner (reviewed in ref. 7 and 8). The oligomerization state of sHSPs can be influenced by the changes in pH,^9^ temperature^10^ as well as by post-translational modifications, such as phosphorylation^11^ that affects their affinity to other proteins and chaperone-like activity.^12^ This relationship between changes in oligomerization and chaperone-like activity has been clearly shown for the human HSPB1 and HSPB5 as well as for the yeast HSP42.^13–15^

While the dynamics of sHSPs oligomerization and oligomers structure were analyzed in detail mainly for archaeal and eukaryotic proteins, the common features of sHSPs seem to be universal in all kingdoms of life (for an extensive review, we refer to ref. 2). The molecules of sHSPs are composed of three structural domains. The central α-crystalline domain (ACD) consisting of about 90-aa is extremely conserved in structure and comprises seven or eight anti-parallel β-strands that form a β-sandwich.^16^ The ACD is flanked by variable N-terminal domain (NTD) and a short C-terminal domain (CTD).^5,14,17–19^ The importance of N- and C-terminal domains for the modulation of sHSP chaperone-like activity by the formation of stress-induced macromolecular assemblies has been previously multiply reported. Being in an oligomeric state, sHSPs recruit the misfolded proteins and prevent their further aggregation.^5,13–15,17,20,21^

The intrinsically disordered N-terminal domain is generally more or less conserved and contains either one or two (W/F)(D/F)PF-like motifs, which are shown to participate in the oligomerization of sHSPs and are required for their chaperone-like activity.^16^ Thus, the removal of the NTD in sHSP26 from *Saccharomyces cerevisiae* led to the formation of di- and tetra-mers which were unable to protect the substrates from thermal denaturation.^22,23^ The removal of 11 N-terminal amino acids of the *Escherichia coli* sHSP also led to the loss of the chaperone-like activity.^24^

The C-terminal domain of many sHSPs also plays an important role in their oligomerization and chaperone-like activity. Generally it contains the conserved V/IXI/V motif necessary for the subunit assembly through interaction with the α-crystallin domain during the oligomerization process.^14,25^ The truncation of V/IXI/V motif or substitution of the isoleucine residues by glycine results in the dissociation of the subunits and thereby in the loss of the chaperone-like activity.^18,25^ In sHSP IbpA from *E. coli*, in addition to the conserved V/IXI/V motif, the arginine 133 in the C-terminus was shown to interact with glutamic acid at position 62 in the α-crystalline domain thereby providing higher order structure formation and playing an important role in the IbpA chaperone function.^24^

In *E. coli*, two sHSP proteins IbpA and IbpB (inclusion body associated proteins), the best characterized bacterial sHSPs, work in strong cooperation^24,26,27^ and belong to the energy-independent part of the proteins quality control network.^28^ These proteins bind partially denatured proteins and prevent their further aggregation thereby facilitating their subsequent refolding by the ATP-dependent DnaK–DnaJ–GrpE and GroEL/GroES chaperone systems in cooperation with the AAA^+^ proteases ClpP and Lon.^10,29–31^*In vitro* studies revealed that both IbpA and IbpB are required to efficiently stabilize denatured proteins in a folding-competent state; however, they play different roles in this process.^10,32^ IbpA abrogates the formation of large aggregates of the substrate simultaneously inhibiting ClpB–DnaK-dependent refolding because of its tight association with polypeptides in aggregates that prevents their processing by the ClpB and DnaK/DnaJ/GrpE chaperones. By the way, IbpB interacts with aggregates *via* IbpA and alleviates the IbpA-mediated inhibitory effect.^32^

*In vitro*, the purified IbpB has been shown to form 2–3 MDa oligomers,^1,33^ while IbpA forms fibril-like structures.^34^ In the mixture of these two proteins, IbpB blocks fibril formation by the IbpA.^24^ The removal of 11 C-terminal amino acids in IbpA abrogated the fibrils formation and the protein was unable to stabilize substrates. In contrast, the N-terminal truncation led to thinner fibrils formation and to the inability to interact with substrates and prevent their aggregation,^24^ apparently suggesting that fibril formation could be an important structural feature of sHSPs involved in its chaperone-like activity.

While in some (often symbiotic) bacteria there are up to 12 different sHSPs proteins,^2^ to the date only few bacterial sHSPs are described. Besides IbpA and IbpB from *E. coli*, four proteins are identified in *Agrobacterium tumefaciens* (HspC, HspL, HspAT1 and HspAT2), and one sHSP in phytopathogen *Xanthomonas*.^19,24,35,36^ Among archaea, genomes of *Deinococcus radiodurans* and *Sulfolobus solfataricus* carry two genes encoding sHsp proteins, while only one small heat shock protein is found in *Methanococcus jannaschii* and *Sulfolobus tokodaii*.^27,37,38^ In contrast, there are several (mostly pathogenic) bacterial genomes that do not encode any sHSPs at all.^2^

While the inactivation of either IbpA or IbpB led to rather moderate effects on the growth and viability of *E. coli* at high temperatures,^39^ the presence of sHSPs in mycoplasmas, microorganisms with significantly reduced genomes, indicates their fundamental importance in the stress resistance of bacterial cells. The phytopathogenic mycoplasma *Acholeplasma laidlawii* contains only one single gene encoding sHSP.^40,41^ Under heat shock conditions, the amount of *Al*IbpA protein in *A. laidlawii* cells increases up to 7% of the total cellular proteins and could appear a key factor that determines the temperature adaptation capacity of this bacterium.^40,41^*In vitro Al*IbpA was shown to exhibit the chaperone-like activity by preventing the DTT- or heat-induced aggregation of insulin^40^ as well as of various cellular proteins from *E. coli* and *A. laidlawii* itself.^42,43^

In the following we unravel the roles of both N- and C-terminal regions of *Al*IbpA for self-oligomerization, substrate binding and the chaperone function regulation. Our data indicate that N-terminus behaves as an auto-inhibitor and activity regulator of the C-terminus which in turn is required for the chaperone function and protein oligomerization in fibrillar form.

## Materials and methods

### Strains and plasmids

*A. laidlawii* PG8 was obtained from the collection of the Institute of Cytology, RAS, and cultivated in Mycoplasma broth with Supplement G (Oxoid) and 1% glucose. *E. coli* XL-10 Gold cells were used for cloning procedures. *E. coli* BL21 (*dcmompThsdS*(r_B_^−^ m_B_^−^)*galλ*(DE3)) was used for proteins overexpression.

Plasmids used in this study were obtained by cloning of the truncated *ibpA* genes into pET15b vector and are listed in Table S1.† pET15b-IbpA that provides the expression of N terminally His_6_-tagged *Al*IbpA was obtained previously.^40^ The truncated and mutant *ibpA* genes were amplified from *A. laidlawii* genomic DNA by using primer pairs as shown in Table S1† to obtain a given plasmid. Sequences of primers used are shown in Table S2.† The pET15b vector was digested with the restriction endonuclease *BamH*I. The digested plasmid and the PCR product were assembled using an isothermal, single-reaction method for assembling multiple overlapping DNA molecules as described previously.^44^ Point mutations leading to the substitution of F11F12 by N11N12 on the N-terminus were introduced by using the QuikChange Site Directed Mutagenesis Kit (Stratagene).

### Purification of His_6_-tagged proteins

*E. coli* BL21(DE3) cells were transformed with plasmids present in Table S1† carrying various *ibpA* genes. The cells were grown at 37 °C in 500 ml of LB medium supplemented with 100 μg ml^−1^ ampicillin. After growth of the culture to an optical density (OD_600_) of 0.8, proteins expression was induced by the addition of IPTG (until final concentration 1 mM). Four hours after induction, cells were harvested by centrifugation and stored at −20 °C.

All proteins with N-terminal His_6_-tag peptide were purified using affinity chromatography on Ni-NTA sepharose gravity-flow columns (Qiagen) following the protocol described previously.^45^*E. coli* cell free extracts were prepared from the frozen cells (see above) by resuspending the cells in disruption buffer DB [50 mM Tris–HCl pH 7.4, 50 mM KCl, 500 mM NaCl, 5 mM MgCl_2_, 1 mM EDTA, 2 mM benzamidine, 1 mM phenylmethylsulfonyl fluoride (PMSF)] and broken by sonication. Unbroken cells and debris were removed after 10 min centrifugation at 8.000 rpm. The cell-free extracts were centrifuged again for 20 min at 15.000 rpm. The supernatant was passed through a 1 ml His-select cartridge column that was pre-equilibrated with washing buffer (50 mM Tris–HCl pH 8.0, 500 mM NaCl, 10 mM imidazole) and unbound proteins were washed out with 15 ml washing buffer. Finally, the affinity-bound proteins were eluted with 8 ml elution buffer (50 mM Tris–HCl pH 8.0, 500 mM NaCl, 500 mM imidazole). All proteins were purified to apparent electrophoretic homogeneity and dialyzed against PBS pH 7.4 containing 100 mM NaCl and used immediately for experiments or stored in ice no more than 4 days. Protein concentration was measured with the Roti-Quant protein assay reagent (Carl Roth) and bovine serum albumin as a standard.

### Chromatography

The analytical size-exclusion chromatography was performed on Superdex 200 10/300 column (Sigma) at flow rate of 0.5 ml min^−1^ on Waters Breeze 2 equipment with the absorbance detection at 280 nm. The protein sample (1 mg ml^−1^) was centrifuged for 5 min at 12 000 rpm to remove any sediment and 100 μl were injected on the column. The running buffer consisted of 50 mM NaCl, 50 mM KCl, 50 mM K_2_HPO_4_ pH 7.4. The apparent molecular weights of proteins were estimated after calibration of the column with following proteins: blue dextran (2000 kDa), β-amylase (200 kDa), alcohol-dehydrogenase (147 kDa), BSA (66 kDa), papain (23,4 kDa), lysozyme (14,3 kDa).

### *In vitro* cross-linking of proteins

The chemical cross-linking by glutaraldehyde (GA) was performed as described in^46^ with modifications. Proteins were dialyzed overnight in a PBS pH 7.4. The reaction mixture consisted of 1.5 μg μl^−1^ of dialyzed *Al*IbpA protein and 0.05% (w/w) freshly prepared solution of glutaraldehyde. The reaction was performed at 25 °C for 20 minutes and then stopped by the addition of 100 mM Tris pH 7.4. The samples were further analyzed by immunoblotting with *Al*IbpA-specific antibodies obtained previously^40^ and developed by using anti-rabbit antibodies conjugated with peroxidase (SigmaAldrich cat. no A0545).

### Pull down assay

For the protein–protein interaction assays, the His_6_-tagged *Al*IbpA proteins (0.4 mg) and bovine insulin (Sigma) (0.4 mg) were diluted in 300 μl of buffer A (50 mM Tris–HCl pH 8.0, 200 mM NaCl) and incubated at 37 °C for 30 min.^45^ Afterwards, the protein mix was loaded to Ni-NTA sepharose (Qiagen) equilibrated by 10 column volumes (10 × 0.2 ml) of buffer A following by washing 4 times by 5 volumes of the same buffer. Proteins were eluted by buffer B (buffer A supplemented with 500 mM imidazole). The samples were collected, precipitated with TCA and separated with 17.5% SDS-PAGE.

### Surface plasmon resonance assay

SPR experiments were performed by using a BIAcore T200 biosensor system (GE Healthcare Life Sciences). The running buffer contained 10 mM Na_2_HPO_4_, 150 mM NaCl, pH 7.4. Purified His_6_-IbpA proteins were immobilized on the Ni^2+^-loaded NTA (nitrilotriacetate) sensor chip to FC2 (flow cell 2) in a volume of 10 μl with a flow rate 10 μl min^−1^ to receive a binding signal of approximately 2000 RU, which corresponds to a surface concentration change of 2 ng mm^−2^. The transcription factor TnrA from *B. subtilis* (His_6_-TnrA20) with deleted protein-binding domain^45^ was injected onto FC1 and served as a control for unspecific interactions. Then the alcohol dehydrogenase (SigmaAldrich, cat. no 55689) at the concentration of 1.0 mg ml^−1^ was loaded on the *Al*IbpA-loaded chip surface pre-washed with 3 mM EDTA. Injection of the protein sample was performed with a flow rate of 10 μl min^−1^ in the running buffer and the response difference (FC2 − FC1) was recorded. To load fresh *Al*IbpA-His_6_ protein on the NTA sensor chip, bound proteins were first removed by injecting 10 μl of 0.5 M imidazol pH 7.0. The data were fitted using the BiaEvaluation and GraphPad Prism software.

### Chaperone-like activity assay

The chaperone-like activity was investigated by measuring the capacity of *Al*IbpA to suppress the heat-shock-induced aggregation of bovine insulin.

Bovine insulin as a model substrate and egg albumine (Sigma, USA) as a control were used in final concentrations 0.5 mg ml^−1^. Final concentrations of mycoplasmal sHSP were 0.5 mg ml^−1^ (*Al*IbpA itself, *Al*IbpA in mixture with insulin). Experiments were carried out at 55 °C for 30 min in total volume of 100 μl (PBS 1×, pH 7.4). Samples (1 μl) were taken every 3 min and light scattering measurements were done with NanoDrop 2000 (Thermo Scientific, USA) at the wave length of 360 nm.

The protein aggregation was also assessed by measuring the fluorescence of the SYPRO Orange, which associates with denatured proteins. The mixtures of various *Al*IbpA proteins (0.4 mg) with insulin (0.4 mg) in total volume of 30 μl were heated in PBS from 10 °C to 95 °C with a temperature increment by 1 °C per 1 min in the presence of 10 μM SYPRO Orange, the fluorescence was detected by using FAM-filter set detection on Bio-Rad CFX96 thermocycler. The data analysis was performed as described in ref. 47 and 48 by determining the temperature leading to fluorescence increase by 50% of maximal signal (*T*_m 50_).

### Transmission electron microscopy

The *Al*IbpA samples were negatively stained on grids coated with collodion films that were discharged in UV (1 min) prior to the application of 5 μl of the protein solution (1–10 μg ml^−1^ in PBS, pH 7.4). The protein solution was blotted off after 2 min, immediately followed by adding and blotting off after 2 min of 3 μl 2% aqueous uranyl acetate. Negatively stained grids were visualized in a Libra 120 electron microscope (Zeiss, Germany) at 25 000–40 000 magnification.

### Bioinformatics

The multiple alignment of proteins amino acid sequences was performed by using Clustal Omega web server^49^ and manually refined. The protein sequences were derived from NCBI database: the nonphotosynthetic alga *Polytomella parva* (Polyt), green photosynthetic alga *Chlamydomonas reinhardtii* (Cr; XP_001703658.1), land plants *Physcomitrella patens* (Physco; BAF36548.1), *Arabidopsis thaliana* (At; NP_192099.1), *Oryza sativa japonica* (Os; Os05g0133100), and *Solanum lycopersicum* (Sl; AAR14689.1), red algae *Porphyra purpurea* (Pp; NP_053864.1), *Porphyra umbilicalis* (Pu; AFC39923.1), and *Pyropia yezoensis* (Py; AGH27579.1), cyanobacteria *Synechococcus elongatus* PCC 7942 (Sy; P0A3F4.1), *Synechocystis* sp. PCC 6803 (Sc; CAA66127.1), and *Escherichia coli* (Ec; CAQ32926.1).

The models of tertiary structures of sHSPs were obtained by using Phyre2 (ref. 50) or I-TASSER^51,52^ servers and also analyzed with in-house developed algorithm.^53^ The superposition of sHSP molecules was obtained with SuperPose online software.^54^

### Statistical analysis

All experiments were performed in biological triplicates with three repeats in each run. The data were analyzed and graphically visualized using GraphPad Prism version 6.00 for Windows (GraphPad Software, USA, https://www.graphpad.com). The comparison with the control has been performed using the non-parametric Kruskal–Wallis one-way analysis of variance test. Significant differences were reported at *p* < 0.05. Densitometry was performed by using the Bio-Rad software for XRS + gel documentation system. The analysis of elution profiles was performed using Waters Breeze 2 software. The analysis of TEM images was based on the shape-based algorithm of globular *vs.* fibrillar structures selection^55^ with subsequent sub-populations quantification using an in-house developed algorithm and software.^56^

## Results

### Analysis of amino acid sequence of *Al*IbpA

**Fig. 1:**
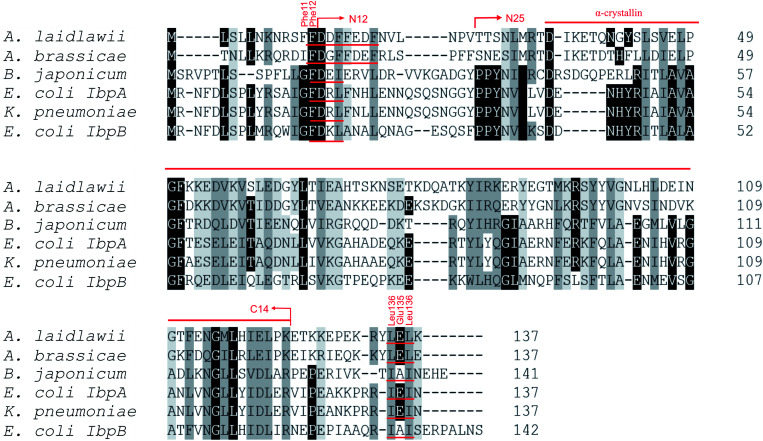
Multiple alignment of amino acid sequences of sHSPs from various bacteria. Amino acids in black represent identical residues, homologous substitutions are shown in grey. Putative (W/F)(D/F) PF and V/IXI/V motifs are underlined. Truncations are shown with arrows.

Most bacterial sHSPs exhibit common conserved structure and consist of α-crystalline domain flanked by variable N-terminal and C-terminal regions.^14,17,18^*Al*IbpA has maximum identity with Hsp20 proteins from various *Clostridia* and *Faecalibacterium* species (45%) and exhibits only 18% and 20% identity with IbpA and IbpB from *E. coli* (*Ec*IbpA and *Ec*IbpB), respectively (Fig. 1, Table S3†). The central α-crystalline domain in *Al*IbpA is highly conserved and it's *in silico*-reconstructed structure demonstrates high similarity with both *Ec*IbpA and *Ec*IbpB despite of the low identity of their amino acid sequences (Fig. S1 and Table S3†). It comprises seven anti-parallel β-strands forming a hydrophobic β-sandwich which could tend to spontaneous oligomerization similarly to other sHSPs.^16^

Both N- and C-terminal regions of *Al*IbpA contain all sHSP-specific motifs, while differ significantly from the well-characterized IbpA and IbpB from *E. coli* (Fig. 1 and S1, Table S3†). In particular, the N-terminus of *Al*IbpA is four amino acid residues shorter compared to that one of *Ec*IbpA and *Ec*IbpB, according to the most probable models obtained with either Phyre2 (ref. 50) or I-TASSER^51,52^ servers. Moreover, it does not exhibit the α-helical structure, albeit containing two tandem (W/F)(D/F)PF-like motifs (Fig. 1). Notably, similar double N-terminal (W/F)(D/F)PF-like motifs with high abundance of Phe-residues are highly conserved among Hsp20 proteins from various *Acholeplasma* spp known to the date (Fig. S2†) that likely appears a specific feature of sHSPs from these bacteria. Since the N-terminal (W/F)(D/F)PF-like motifs are required for the oligomerization and for the chaperone-like activity of sHSPs,^16^ their existence in the N-terminus of *Al*IbpA suggests that the protein might implement chaperone-like activity in the same manner like the other sHSPs do.^16^ Next, the C-terminus of *Al*IbpA contains LEL amino acid residues apparently constituting the conserved V/IXI/V motif, which was shown to be necessary for the interaction with the α-crystalline domain of *Ec*IbpA during oligomerization, as its truncation completely abolished the chaperone-like activity of the protein.^24^ This fact allows assuming that *Al*IbpA might exhibit chaperone-like activity while being in the oligomeric form.

For further assessment of the roles of both N- and C-termini of *Al*IbpA in the oligomerization and thereby also in the chaperone-like activity, various truncated recombinant proteins have been constructed. The removed parts from either N- or C-termini are shown in Fig. 1. In particular, *Al*IbpAΔN12 lacks the first 12 amino acid residues and carries the truncated first (W/F)(D/F)PF-like motif, which is probably required for the oligomerization and thus also for the chaperone-like activity. In *Al*IbpAN11N12 both F11F12 are substituted by N11N12 leading to the damage to the motif while keeping the complete N-terminus length. *Al*IbpAΔN25 lacks both (W/F)(D/F)PF-like motifs. In order to understand the role of the C-terminal LEL motif, 14 amino acids flanking the α-crystalline domain from the C-terminus were removed by obtaining *Al*IbpAΔC14.

It has been shown previously^24^ that the IEI-motif is necessary for the interaction with the α-crystalline domain of *Ec*IbpA during oligomerization *via* binding with glutamic acid at position 62 in the α-crystalline domain. In *Al*IbpA this position corresponds to lysin according the alignment (Fig. 1), and both electrostatic and hydrophobic interactions are possible. Therefore the *Al*IbpASEP with L134 and L136 replaced by polar amino acids S134 and P136 (substitution of the LEL motif by SEP) have been constructed to keep the length of the C-terminus (Fig. 1). Thus, the first leucine residue was replaced by polar serine, while the second leucine residue was replaced by proline to obtain the turn of the chain and provide a steric barrier for the interaction of C-terminus with the α-crystallin.

Additionally, *Al*IbpAΔN12C14, *Al*IbpAΔN25C14, *Al*IbpASEPΔN12 and *Al*IbpASEPΔN25 with truncated both N- and C-termini have been constructed. The recombinant wild-type and the truncated *Al*IbpA were expressed in *E. coli* and purified to the apparent electrophoretic homogeneity (Fig. S3†).

### Evaluation of oligomers formation

It has been reported earlier that sHSPs exhibit their chaperone-like activity while being in huge oligomers consisting typically between 9 and 48 subunits.^1,5,6^ Therefore the oligomerization of *Al*IbpA has been analyzed *in vivo* at various temperatures by chemical cross-link.

**Fig. 2:**
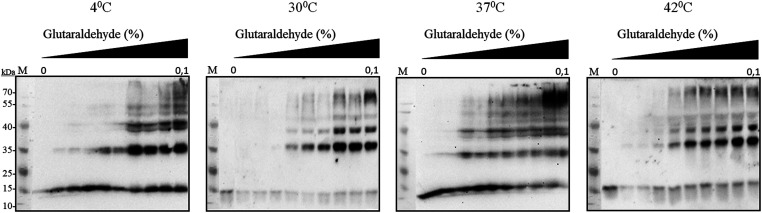
*In vivo* chemical cross-link of native *Al*IbpA. *A. laidlawii* cells at the late exponential growth phase were incubated for 1 h at various temperatures as indicated and treated with glutaraldehyde. The crude cell extracts were prepared and analyzed by immunoblotting using anti-*Al*IbpA antibodies.

**Fig. 3:**
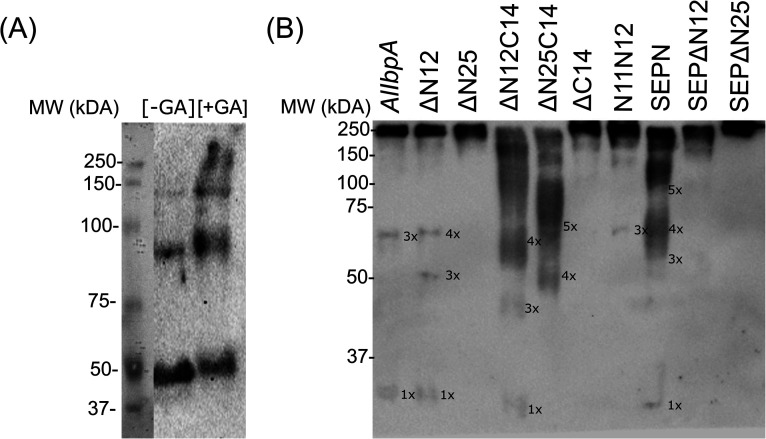
*In vivo* (A) and *in vitro* (B) chemical cross-link of full-length recombinant *Al*IbpA-His_6_ (A) and its various truncated versions (B). The synthesis of *Al*IbpA-His_6_ by *E. coli* BL21 pET-IbpA cells was induced by IPTG and after 2 h of induction cells were treated for 1 h with glutaraldehyde at 37 °C and the crude cell extracts were prepared. Alternatively, the purified full-length *Al*IbpA or *Al*IbpA proteins with different N- and C-terminal truncations (50 ng of each protein) were incubated 1 h at 37 °C and then cross-linked with glutaraldehyde. Then the proteins were separated in 10% SDS-PAGE and analyzed by immunoblotting using anti-*Al*IbpA antibodies.

*A. laidlawii* were grown in liquid medium until the late exponential growth phase. Then the aliquots of culture were incubated for 1 h at various temperatures as indicated, fixed with glutaraldehyde and analyzed with immunoblotting. These data clearly indicated that *Al*IbpA is present in the cells as aggregates/multimers regardless of the temperature (Fig. 2). Next, both *in vivo* and *in vitro* cross-link of recombinant full-length *Al*IbpA confirmed that the protein is present as oligomers at 37 °C (Fig. 3).

Surprisingly, the removal of neither N- nor C-termini of *Al*IbpA did not abolish the oligomerization, while the double removal of both N- and C-termini led to the decrease of the oligomerization degree (Fig. 3B). Similarly, the removal of 12 N-terminal amino acid residues from the protein with mutated C-terminal LEL-motif abrogated the large multimers formation, while di-, tri- and tetramers were still observed. These data suggest that the protein forms oligomers by either N- or C-terminus, in contrast to *Ec*IbpA, which was shown to be in low-oligomeric state when either N- or C-terminus had been removed.^24^

### The role of N- and C-termini in substrates binding and chaperone-like activity of *Al*IbpA

To evaluate the importance of N- and C-termini of *Al*IbpA for the substrate binding, the interaction of various proteins with *Al*IbpA has been assessed *in vitro* with the Surface Plasmon Resonance (SPR). Only alcohol dehydrogenase (ADH) and in a lesser extent the bovine insulin (SigmaAldrich cat no. I6634) were able to interact with *Al*IbpA in SPR assay (see Fig. S4†).

**Fig. 4:**
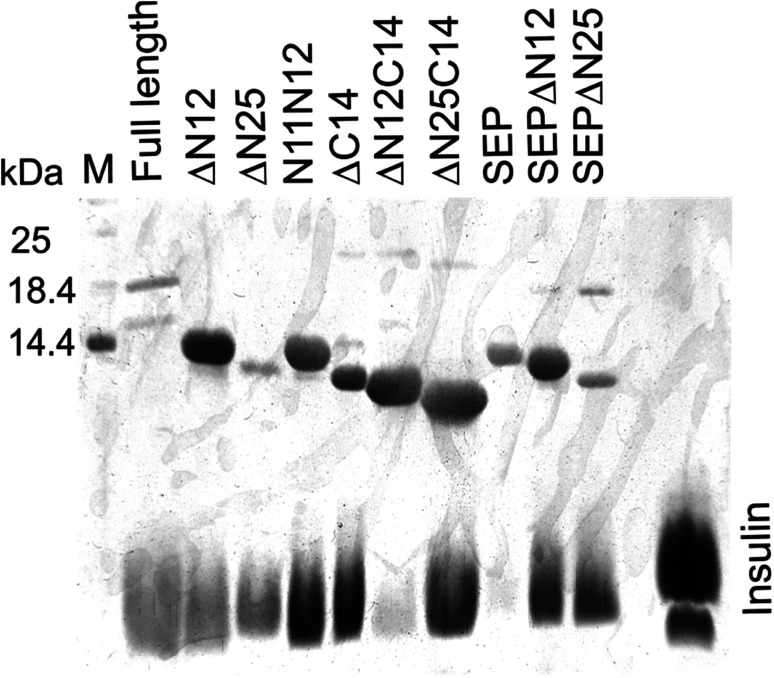
Interaction of truncated *Al*IbpA proteins with insulin *in vitro* assessed with pull down assay. Proteins were mixed in PBS in weights ratio of 1 : 1 and after 30 min incubation at 37 °C were purified on Ni-NTA agarose and elution fractions were separated with 17.5% SDS-PAGE.

To further characterize the functional role of N- and C-termini of *Al*IbpA, its chaperone-like activity has been also evaluated *in vitro*. It has been shown previously that *Al*IbpA demonstrates the chaperone-like activity *in vitro* by preventing the temperature-induced denaturation of the bovine insulin.^40^ Taking into account the fact that the denaturation temperature (*T*_m 50_) of ADH and other proteins exceeded that of *Al*IbpA, the bovine insulin has been chosen as a model substrate for the chaperone-like activity assays (see Table S4† for values). For that, the *T*_m 50_ of insulin in the presence of either full-length or truncated *Al*IbpA proteins was determined by using the fluorescent SYPRO-orange dye as described in Materials and methods (see Table S4† for values). Additionally, the interaction of *Al*IbpA proteins with insulin was analyzed by using pull-down assay. For that, *Al*IbpA proteins and insulin were mixed in 1 : 1 weights ratio and after 30 min of incubation at 37 °C were purified on Ni-NTA agarose followed by SDS-PAGE analysis (Fig. 4, Table 1).

**Table 1:** The chaperone-like activities, substrate binding and oligomerization capabilities of full-length and truncated *Al*IbpA proteins^a^

*Al*IbpA variant	Protein structure	bΔ*T*_m 50_, °C		cInsulin binding	dADH binding *K*_D_, μM	eDistribution of various oligomeric fractions, %		
		eIbpA	fIbpA + insulin			I peak	II peak	1×-4×-mers
Full length	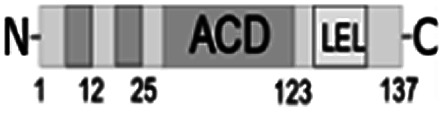	0^g^	+5 ± 0.8	++	1.6 ± 0.3	23 ± 10.3	74 ± 17.8	3 ± 0.2
ΔN12	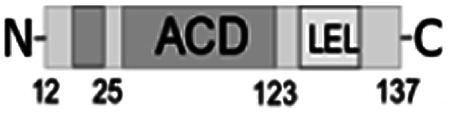	+3 ± 0.3*	+10 ± 2.3*	+	3.5 ± 1.1	88 ± 17.1	10 ± 1.1	3 ± 0.1
ΔN25	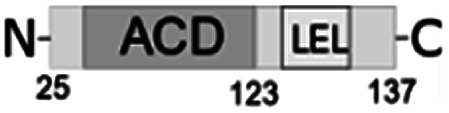	+3 ± 0.4*	+10 ± 2.1*	++	2.2 ± 0.7	77 ± 21.3	12 ± 2.4	11 ± 2.1
N11N12	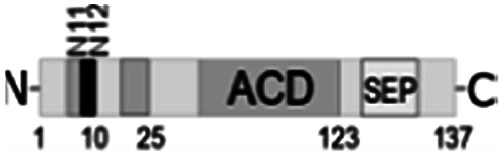	+5 ± 0.8*	+11 ± 2.4*	++	0.5 ± 0.1	8 ± 1.7	88 ± 12.2	4 ± 0.7
ΔC14	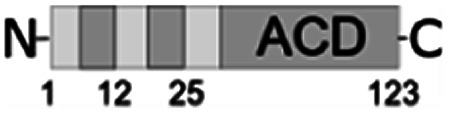	−7 ± 0.6*	−3±1.0*	++	0.7 ± 0.1	7 ± 4.1	86 ± 23.4	7 ± 2.3
ΔN12C14	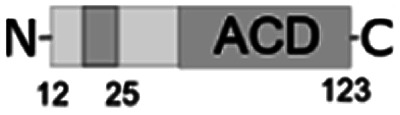	−5 ± 0.7*	−6±1.2*	—	NA	1 ± 0.1	86 ± 22.8	14 ± 10.3
ΔN25C14	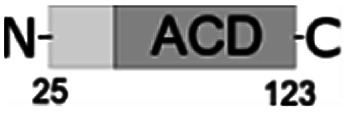	−13 ± 2.2*	−9±1.1*	+	NA	1 ± 0.2	50 ± 13.5	49 ± 10.3
SEP	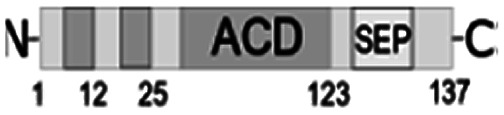	−2 ± 0.1	+2 ± 0.2*	—	NA	18 ± 4.4	56 ± 7.8	26 ± 5.9
SEPΔN12	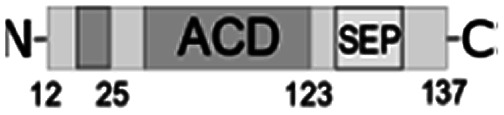	−1 ± 0.3	+3 ± 0.5	+	7.2 ± 1.4	2 ± 0.8	59 ± 10.1	39 ± 9.9
SEPΔN25	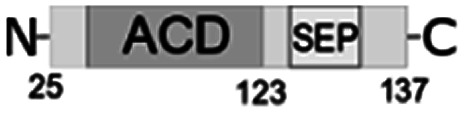	+1 ± 0.2	+5 ± 0.9	++	3.2 ± 0.5	69 ± 15.5	23 ± 3.2	8 ± 2.0

a * – denotes statistically significant difference with full-length *Al*IbpA.

b Determined by measuring the sypro-orange fluorescence.

c Determined as ratio of *Al*IbpA and insulin bands density on *in vitro* pull-down assay SDS-PAGE (see ESI Fig. S5): “−” – no interaction, “+” – 1 : 1 ratio, “++” 1 : 2 and higher.

d Calculated from the ADH-to-*Al*IbpA surface binding curve slopes obtained with SPR (NA – not available because of no interaction).

e Calculated as fractions of areas under the peaks on the elution profile.

f The *T*_m 50_ value of full-length *Al*IbpA is accepted as zero.

g The *T*_m 50_ value of insulin in absence of full-length *Al*IbpA is accepted as zero.

**Fig. 5:**
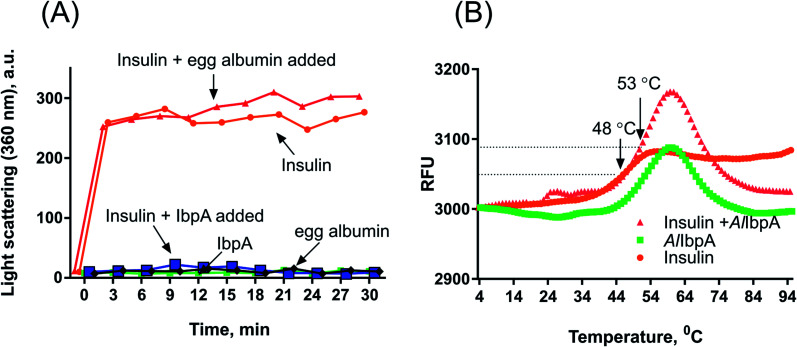
The chaperone-like activity of recombinant *Al*IbpA. The *Al*IbpA was mixed with insulin in PBS in weight ratio of 1 : 1. (A) Proteins mixture was incubated for 30 min at 55 °C with measuring the light scattering of the mixture at 360 nm each 3 min. (B) Proteins mixture was heated in PBS from 10 °C to 95 °C with a temperature increment by 1 °C per 1 min in the presence of 10 μM SYPRO Orange and the fluorescence was detected by using FAM-filter set. *T*_m 50_ was calculated as the temperature leading to fluorescence increase by 50% of maximal signal.

Despite of weak interaction observed by the SPR, the insulin was co-eluted with the full-length *Al*IbpA (Fig. 4, lane 1) suggesting the interaction of proteins under conditions used. In agreement with earlier data, *Al*IbpA was able to prevent the temperature-induced denaturation of the bovine insulin when measuring either the light scattering of proteins mixture during 30 min at 55 °C (Fig. 5A) or the fluorescence of the SYPRO Orange during proteins mixture heating from 10 °C to 95 °C (Fig. 5B). The *T*_m 50_ of insulin was increased by 5 °C (from 48 °C to 53 °C) in presence of *Al*IbpA (Fig. 5B) confirming the presence of chaperone-like activity.

**Fig. 6:**
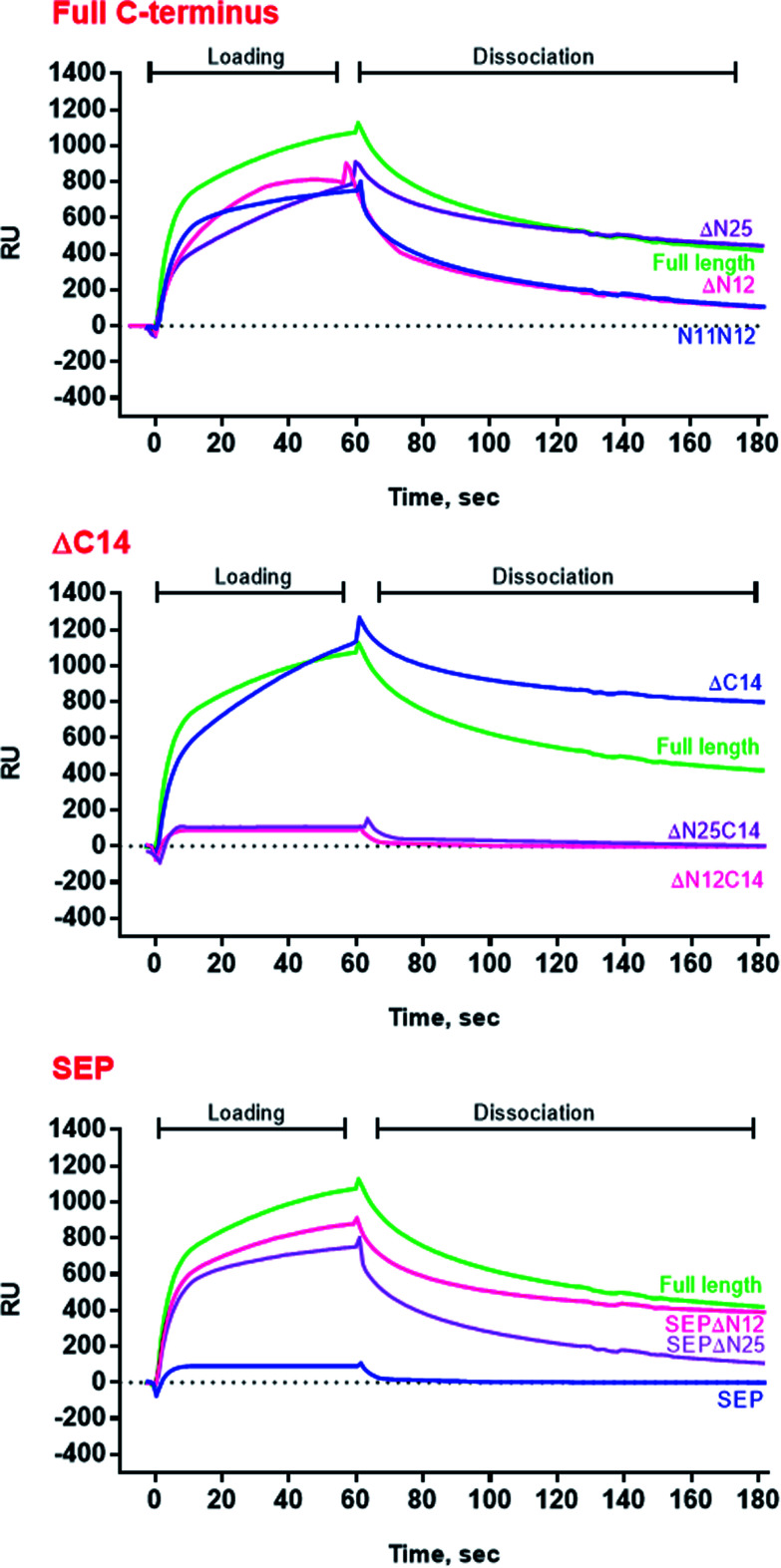
SPR analysis of alcohol dehydrogenase interaction with full-length, mutated and truncated *Al*IbpA proteins. ADH was loaded onto the chip surface with immobilized various *Al*IbpA proteins as indicated. RU-resonance units.

Next the chaperone-like activity and substrate binding capacity of various truncated *Al*IbpA proteins has been evaluated (Table 1). The removal of either 12 or 25 amino acids from the N-terminus as well as the substitution of F11F12 by N11N12 led to the increase of *T*_m 50_ of the protein mixture by 5 °C compared to the full-length protein, while interaction with both insulin and ADH was not affected (Fig. 4 and 6, Table 1). These data suggest that (W/F)(D/F)PF-like motifs are apparently not required for the interaction with substrate proteins as well as for the protein stabilization. Moreover, N-terminus seems to suppress the chaperone-like activity of the full-length protein.

The deletion of the C-terminus significantly decreased the temperature stability of *Al*IbpA itself and its mixture with insulin (Table 1). Nevertheless, both insulin and ADH were still able to bind with *Al*IbpAΔC14, apparently, interacting with its N-terminus, while no protection of the substrate protein from the heat-induced denaturation could be observed (Fig. 4 and 6, Table 1). The removal of both 12 N-terminal and 14 C-terminal amino acids completely abrogated the interaction of *Al*IbpA with both insulin and ADH and also no denaturation prevention could be observed. *Al*IbpA lacking 25 N-terminal and 14 C-terminal amino acids was characterized with the lowest stability and chaperone-like activity. Interestingly, some binding with insulin could be detected, probably by its direct interaction with the α-crystalline domain (Fig. 4).

Next, the substitution of the LEL motif by SEP did not affect significantly the stability of the protein, while abolished both the chaperone-like activity of *Al*IbpASEP and its interaction with both insulin and ADH (Fig. 4 and 6, Table 1). Interestingly, the stability, substrate binding and chaperone-like activity of both *Al*IbpASEPΔN12 and *Al*IbpASEPΔN25 increased in comparison with *Al*IbpASEP and were comparable with those of the full-length protein while remaining significantly lower than in *Al*IbpAΔN12 and *Al*IbpAΔN25.

Taken together these data clearly demonstrate that both N- and C-termini determine the interaction of *Al*IbpA with substrate proteins. In particular, the C-terminus seems to be responsible for the chaperone-like activity of the protein, while the N-terminus could play a regulatory role.

### N-terminal domain provides formation of the globular structure and represses fibril formation

**Fig. 7:**
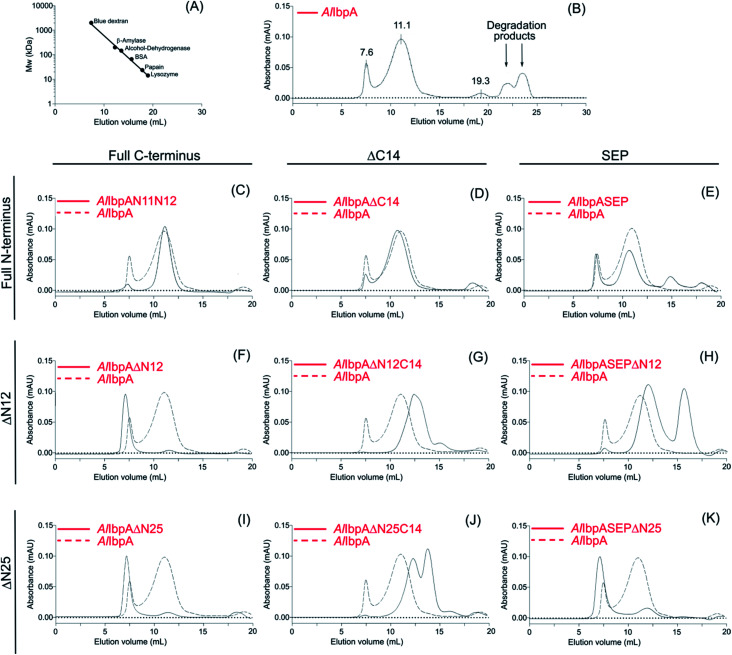
Size-exclusion chromatography of full-length, mutated and truncated *Al*IbpA proteins.

Results of the chaperone-like activity (Table 1) revealed that the interaction with substrates differs between the full-length and the truncated *Al*IbpAs regardless of their oligomerization leading to the assumption of inter-molecular alternations in the oligomer structure. To corroborate this hypothesis, the oligomerization level of both full-length and all truncated modifications of *Al*IbpA proteins have been assessed with size-exclusion chromatography in a thermostatic chamber at 30 °C (Fig. 7).

**Fig. 8:**
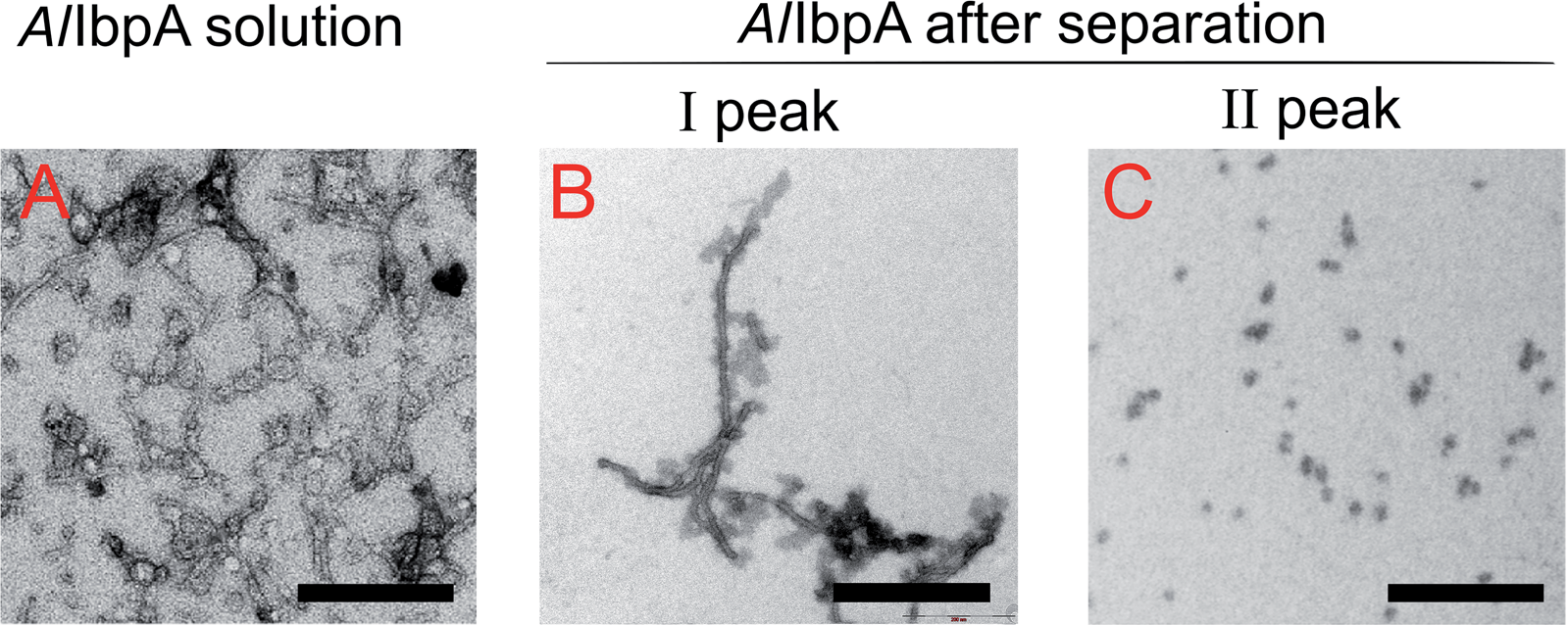
Transmission electron microscopy of cross-linked full-length *Al*IbpA before (A) and after separation on size-exclusion column (B and C). I and II – first and second peaks, respectively, on *Al*IbpA elution profile shown on Fig. 7. The bar corresponds to 200 nm.

The full-length *Al*IbpA was eluted in two main peaks at 7.6 ml and 11.1 ml corresponding to the oligomers of ∼1700 kDa and ∼400 kDa, respectively (Fig. 7B). First two peaks corresponding to the high molecular weight oligomers of the protein were harvested and immediately subjected to the *in vitro* cross-linking with glutaraldehyde, negatively stained on grids and analyzed with transmission electron microscopy (Fig. 8). As indicated by the microscopy data, the full-length *Al*IbpA is represented by the heterogeneous mixture of both globular and fibrous structures. After gel-filtration, in the first peak the mixture of fibrils and protein agglomerates was eluted, while the second peak corresponds to globules consisting of apparently 24 monomers (estimated molecular weight 451 kDa).

**Fig. 9:**
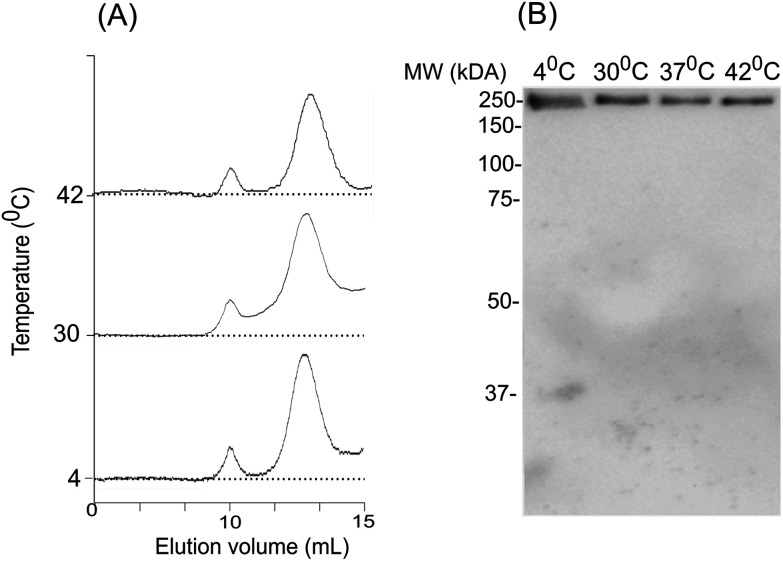
The effect of temperature on oligomerization state of *Al*IbpA. Size-exclusion chromatography of the full-length *Al*IbpA-His_6_ protein has been chemically cross-linked at 4 °C, 37 °C and 42 °C and analyzed with size-exclusion chromatography (A) and SDS-PAGE (B).

To assess the temperature-dependent inter-molecular rearrangements of sHSP oligomers, the full-length *Al*IbpA was cross-linked after 1 h incubation at different temperatures (4 °C, 30 °C and 42 °C) before the chromatography, to avoid the inter-molecular reorganizations during the gel-filtration. The elution profiles of the protein did not differ significantly from the untreated one suggesting relative stability of *Al*IbpA oligomeric forms (Fig. 9A). The SDS-PAGE analysis of cross-linked proteins also confirmed that recombinant protein existed as huge oligomers independently of the temperature (Fig. 9B). These data suggest that the full-length *Al*IbpA in solution is represented by a mixture of 24-mer globules of ∼400 kDa and high-molecular weight fibrils/agglomerates with about 3 : 1 ratio regardless of the temperature (Table 1).

**Fig. 10:**
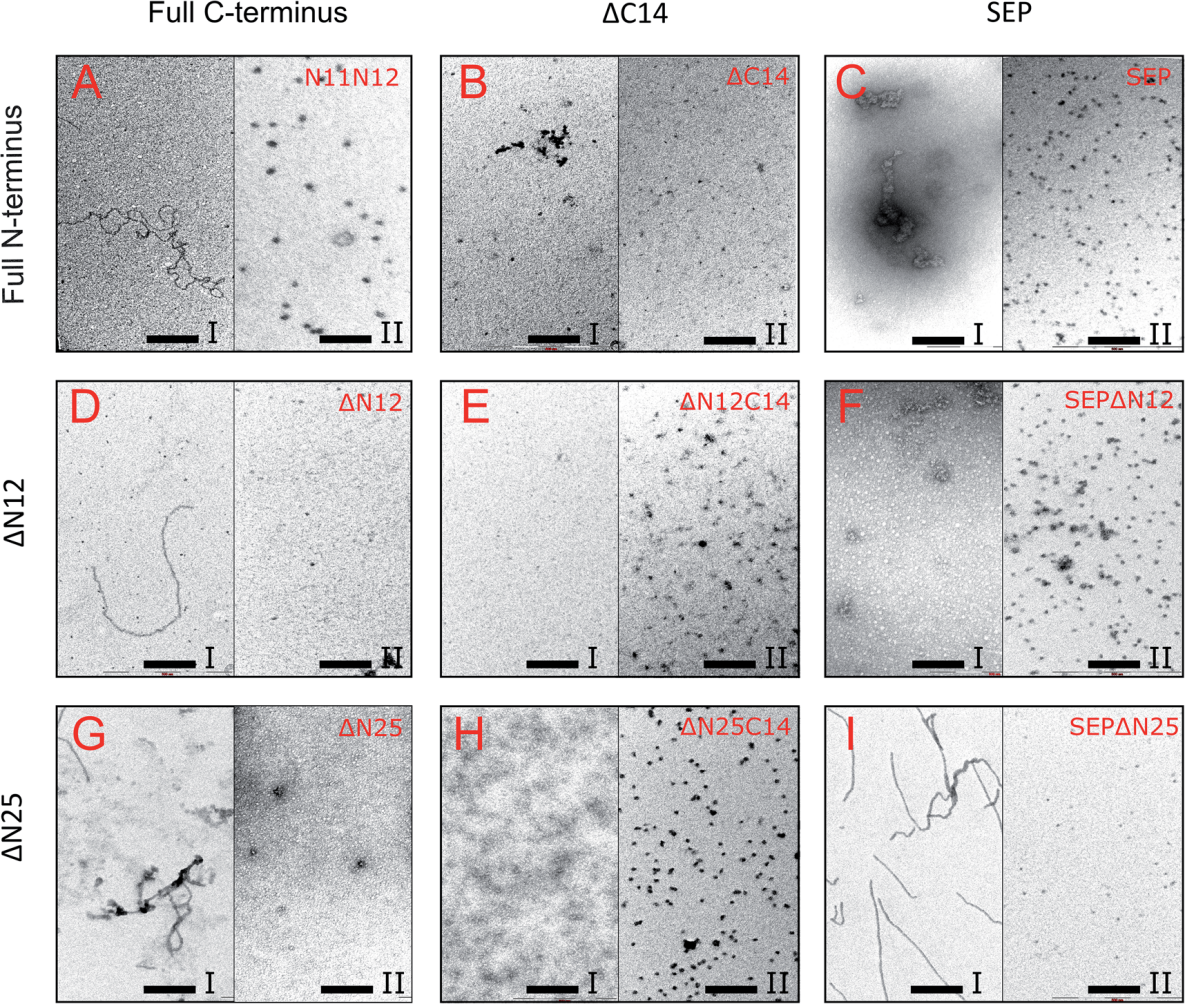
Transmission electron microscopy of mutated and truncated *Al*IbpA proteins after separation on size-exclusion column. I and II – first and second peaks, respectively, shown on Fig. 7 were collected and immediately subjected to cross-linking with 0.05% glutaraldehyde. 5 μl of solution were transferred to the grids and negatively stained. The bar corresponds to 200 nm.

Next, the truncated proteins were analyzed also with transmission electron microscopy in order to discover their oligomeric structure (Fig. 10). Either removal of 14 amino acids from the C-terminus or truncation of the LEL motif barely affected the ratio of globules and huge multimers in the protein solution (Fig. 7D and E), while no fibrils could be observed with both *Al*IbpASEP and *Al*IbpAΔC14 being present as agglomerates in the first peak fraction (Fig. 10B and C). In marked contrast, while the truncation of the first two F-residues did not affect the globules formation (Fig. 7C and 10A), the removal of either 12 or 25 amino acid residues from the N-terminus of protein completely abolished the globules formation and the protein was present predominantly as long fibrils (Fig. 7F, I and 10D, G). The removal of both N- and C-termini of the protein led to a significant decrease of the oligomers molecular weight to ∼200 kDa (Fig. 7G, J and 10E, H). Similarly, in agreement with *in vitro* cross-link data (Fig. 3B), the removal of 12 N-terminal amino acid residues and the truncation of the LEL-motif also suppressed the oligomerization of the *Al*IbpAΔN12C14 (Fig. 7G and 10E). Remarkably, after removal of 25 N-terminal amino acid residues and mutation in the LEL-motif *Al*IbpASEPΔN25 could be observed in form of fibers (Fig. 7K and 10I). Apparently, in this case either the α-crystalline domain tends to form oligomers by unspecific hydrophobic interactions, or other C-terminal motif also provides the fibrils formation in absence of functionally active LEL-motif.

These data clearly demonstrate that both N- and C-termini in *Al*IbpA participate in the protein oligomerization. The C-terminus seems to be necessary for the fibrous form formation, while the N-terminus seems to be responsible for the globules formation and in turn apparently acts as a suppressor of the transition into fibrous form.

## Discussion

Small heat shock proteins (sHSPs) represent the first line of cellular defense against stress-induced protein aggregation, and their tertiary structures are nearly identical in various organisms from bacteria to mammals. Despite significant variations between amino acid sequences, these proteins consist of the central α-crystalline domain flanked with N- and C-terminal regions.^5,14,17–19^ While the main role for substrates binding and prevention of their aggregation likely belongs to the central α-crystalline domain, the removal of either N- or C-terminal domains generally leads to the loss of the chaperone-like activity, suggesting their necessity for the sHSPs functionality.^18,24,25,57^ Nevertheless, exact mechanisms of how either N- or C-terminal domains regulate the activity of the protein remains questionable. In this work we attempted to unravel the functional role of both N- and C-terminal domains in sHSP *Al*IbpA from *Acholeplasma laidlawii*, a ubiquitous phytopathogen of the genus Mollicutes.

The Mollicutes represent simplest bacteria characterized by a complete lack of cell walls and drastically reduced genome size. Among them, presumably only those species that are able to survive in the environment carry the gene encoding for the sHSP-like protein with classical α-crystalline domain in the genomes (Fig. S2†) suggesting that these proteins are essential for the survival of mycoplasmas under environmental conditions.^40^*Al*IbpA was the first well-described mycoplasmal sHSP isolated from the free-leaving mycoplasma *Acholeplasma laidlawii*.^40,41^ Under the heat-shock conditions, *Al*IbpA becomes the one of the most abundant proteins in *A. laidlawii* confirming its importance for the stress resistance of this bacterium, although the deletion of sHSP genes in *E. coli* did not drastically affect the viability of the cells at elevated temperatures.^39^

The structural organization of *Al*IbpA is similar to other sHSPs and includes the central α-crystalline domain flanked with N- and C-termini (Fig. 1). The N-terminus of the protein contains double (W/F)(D/F)PF-like motif (Fig. 1), which was previously shown to be involved and even required for their oligomerization and chaperone-like activity of other sHSPs.^12,16,22,24^ The truncation of the N-terminus generally abrogates both the interaction with substrates proteins and the oligomerization of sHSPs, leading to only dimers formation, particularly for the IbpA from *E. coli*^24^ and for the HSP26 from *S. cerevisiae*.^22,23^ In marked contrast, the deletion of either 12 or 25 amino acid residues from the N-terminus of *Al*IbpA or partial truncation of the first (W/F)(D/F)PF-like motif affected neither oligomerization (Fig. 3B) nor substrates binding (Fig. 4 and 6) of *Al*IbpAΔN12, *Al*IbpAΔN25 and *Al*IbpAN11N12. Moreover, the protein stability and capability to prevent the temperature-induced aggregation of insulin even increased in comparison with the full-length protein (Table 1). Further analysis of the oligomerization revealed that both *Al*IbpAΔN12 and *Al*IbpAΔN25 are present predominantly in fibrous form (Fig. 7F, I and 10D, G), while the full-length protein represents a mixture of 24-mer globules, fibrils and protein agglomerates with the prevalence of globules (Fig. 7B and 8). Similarly, *Al*IbpAN11N12 is present predominantly as globules consisting of apparently 24 monomers with low fraction of long fibrils as indicated by both gel-filtration and TEM data (Fig. 7C and 10A). Of note, one could assume that the presence of globules on TEM image of the first elution peak of full-length *Al*IbpA (Fig. 7I peak) might be a consequence of the fast reorganization of the protein quaternary structure in contrast to the truncated versions of *Al*IbpA which are predominantly in either fibrillar or globular state.^39^

At the C-terminus of *Al*IbpA the conserved LEL-motif participating in oligomerization and chaperone-like activity of sHSPs has been identified (Fig. 1).^14,18,25^ The removal of the C-terminus of *Al*IbpA completely abrogated the chaperon-like activity of *Al*IbpAC14, while, in contrast to *Ec*IbpA, its interaction with insulin and ADH could nevertheless be observed (Fig. 4 and 6, Table 1). Only double removal of both N- and C-termini completely abrogated both the chaperon-like activity and the substrate proteins binding, suggesting that the N-terminus also participates in the substrate binding, while the chaperon-like activity is apparently provided by the C-terminus only. From the structural point of view, *Al*IbpAΔC14 was observed predominantly as globules (peak II) or their aggregates (peak I), while simultaneous truncations of both N- and C-termini led to the low-level oligomerization of *Al*IbpAΔN12C14 and *Al*IbpAΔN25C14 with the formation of di- and tetramers (Fig. 3B and 7G, J) likely due to the α-crystalline self-oligomerization.

It has been shown previously that the purified *Ec*IbpA and *Ec*IbpB from *E. coli* can be also present as fibrils and huge agglomerates, respectively,^1,33,34^ while in their mixture *Ec*IbpB blocks the fibril formation by *Ec*IbpA.^24^ Therefore, it could be speculated that the N-terminal extension of *Al*IbpA acts as autoinhibitory motif suppressing the fibril formation, similarly as *Ec*IbpB abrogates the fibrillation of *Ec*IbpA (Fig. 7 and 10) while also negatively regulating the chaperone-like activity of the protein (see Δ*T*_m 50_ data on Table 1). Consequently, the removal of the N-terminus could open the α-crystalline domain for the interaction with substrates.

The speculative explanation of fibrils formation by *Al*IbpAΔN12 and *Al*IbpAΔN25 could be the following. The globular form of sHSPs is considered as the inactive form of sHSPs in the cell (used to store the protein) in which a spatial access to the α-crystalline for the interaction with both substrates and the C-terminus is limited.^58^ Under stress conditions, the rearrangement of subunits occurs, thereby opening the α-crystalline for the interaction with the C-terminal LEL motif of the neighboring subunit of *Al*IbpA. Apparently, in the presence of two (W/F)(D/F)PF-like motifs in *Al*IbpA preferential globular structure formation governed by the hydrophobic (W/F)(D/F)PF-like motifs in full-length protein blocking the fibrils formation and substrates binding, could be hypothesized. This assumption fits well with more pronounced chaperone-like activity of *Al*IbpAΔN12 and *Al*IbpAΔN25 (see Table 1). By the way, *Al*IbpAN11N12 appeared also as globules (Fig. 7C and 10A), while more pronounced chaperone-like activity of protein could be observed in comparison with the full-length protein (Table 1). Apparently, the truncation of only one (W/F)(D/F)PF-like motif appears insufficient for the opening of the α-crystalline domain for the interaction with the C-terminus and fibrils formation, while the substrate binding is less limited (Fig. 4 and 6).

Next, to confirm the role of the C-terminal LEL-motif for the *Al*IbpA functions, LEL was substituted by SEP. The resulting *Al*IbpASEP was neither able to bind insulin and ADH nor able to prevent the aggregation of insulin (Fig. 4 and 6, Table 1). Furthermore, *Al*IbpASEP appeared in aggregates on the first peak (Fig. 10C), that is in agreement with earlier reported C-terminal IXI motif in sHSPs interaction with a hydrophobic groove on the α-crystalline domain of a neighboring dimer thereby facilitating the formation of higher-order oligomers.^14,25^ Altogether these data confirm the necessity of the LEL-motif for the fibrils formation and thereby also for the chaperone-like activity of *Al*IbpA similarly to other sHSPs.^14,18,25^ In the second peak the globules of *Al*IbpASEP were observed (Fig. 10C) supporting the hypothesis that the N-terminus is responsible for the globular structure formation.

Nevertheless, any amino acids besides the LEL motif on the C-terminus seem to be required for the protein stabilization, since the substitution of LEL by SEP did not reduce the protein stability (Table 1). Moreover, the removal of the N-terminal amino acids from the *Al*IbpASEP suppresses the mutation of the LEL-motif and restores the insulin binding and the chaperone-like activities of the resulting proteins that appears even more pronounced for *Al*IbpASEPΔN25 than for *Al*IbpASEPΔN12 (Fig. 4, Table 1). In marked contrast, *Al*IbpASEPΔN12 was not able to form high-order oligomers, while *Al*IbpASEPΔN25 was present as fibrils (Fig. 3, 7H, K and 10I). These data suggest that both N-terminal (W/F)(D/F)PF-like motifs are involved in the globular structure formation, allowing fine-tuned activation of the protein depending on the stress force. From the other side, not only the C-terminal LEL motif but also other amino acid residues are involved in fibrils formation by *Al*IbpA, in contrast to other sHSPs, confirming that the *Al*IbpA activity regulation is governing by complex intramolecular mechanisms thereby providing the viability of *A. laidlawii* under environmental conditions.

## Conclusions

Taken together, our data demonstrate non-trivial features of the N-terminal domain of sHSP *Al*IbpA from *A. laidlawii*. We suggest that the N-terminus provides the formation of approximately 24-mer globules of *Al*IbpA while also behaves as an autoinhibitor and activity regulator of the C-terminus. In turn, the C-terminus is required for the chaperone-like activity and protein oligomerization into the fibrous form. Furthermore, our data (with the exception of *Al*IbpAN11N12) show clear association of the fibrils formation by *Al*IbpA and its ability to prevent the temperature-induced aggregation of insulin (see Table 1, Fig. 7 and 10). Thus, while the full-length protein is present as a mixture of agglomerates, fibrils and globules demonstrating moderate chaperone-like activity on insulin, the stability and activity of truncated *Al*IbpA versions appearing as fibrils appears significantly higher. Thus, in contrast to *E. coli* sHSPs IbpA and IbpB which work in strong cooperation and thus apparently regulate their activity, the *Al*IbpA represents a first sHSP20 protein where the competition between N- and C- termini governs the shift of the protein quaternary structure to either fibrous or globular form thereby representing the molecular mechanism of the *Al*IbpA function regulation.

## Author contributions

Conceptualization, A. K. and I. V.; methodology, A. K., V. Ch. and I. V.; software, M. B.; validation, L. Ch., A. K. and I. V.; formal analysis, I. V.; investigation, L. Ch.; resources, A. K. and I. V.; data curation, M. B.; writing—original draft preparation, A. K. and I. V.; writing—review and editing, A. K., I. V. and M. B.; visualization, L. Ch., A. K. and I. V.; supervision, A. K. and I. V.; project administration, A. K. and I. V.; funding acquisition, I. V.

## Funding

This research was funded by the Russian Science Foundation (project no. 17-74-20065 to Innokentii Vishnyakov) while being performed in the framework of the Russian Government Program of the Competitive Development of Kazan Federal University (Liliya Chernova and Airat Kayumov). The APC was funded by the Russian Science Foundation (project no. 17-74-20065 to Innokentii Vishnyakov).

## Conflicts of interest

The authors declare no conflict of interest.

## Supplementary Material

RA-010-C9RA10172A-s001
