# Supplementary material for: N- and C-terminal regions of the small heat shock protein IbpA from Acholeplasma laidlawii competitively govern its oligomerization pattern and chaperone-like activity
Source: RSC Adv. 2020 Feb 26;10(14):8364–76. doi: 10.1039/c9ra10172a (PMC9050003; doi:10.1039/c9ra10172a)
Supplement: RA-010-C9RA10172A-s001 [file RA-010-C9RA10172A-s001.pdf]

Electronic Supplementary Material (ESI) for RSC Advances.
This journal is © The Royal Society of Chemistry 2020

Supplementary material

N- and C-terminal regions of the small heat shock protein IbpA from *Acholeplasma laidlawii* competitively govern its oligomerization pattern and chaperone-like activity

Liliya S. Chernova[1,2], Mikhail I. Bogachev[3], Vitaly V. Chasov[1], Innokentii E. Vishnyakov[2,4*,‡] and Airat R. Kayumov[1,2,*,‡]

[1]Kazan Federal University, Kremlevskaya 18, 420008, Kazan, Russia.
E-mail: kairatr@yandex.ru
Tel.: +7-843-233-7802

[2]Institute of Cytology, Russian Academy of Sciences, Tikhoretsky ave. 4, 194064 St-Petersburg, Russia.
E-mail: innvish@gmail.com
Tel.: +7-812-297-0328

[3]Biomedical Engineering Research Centre, St. Petersburg Electrotechnical University, St. Petersburg, Russia

[4] Peter the Great St.Petersburg Polytechnic University, Polytechnicheskaya, 29, 195251, St-Petersburg, Russia

[‡] The authors contributed equally to this manuscript as senior authors

Table S1. Plasmids used in this study

Plasmid	Description	Primers used for *AlibpA* amplification	Source
pET15b	Expression vector	-	Novagene
pET15b IbpA	Overexpression of His-tagged IbpA in *E.coli*	-	Vishnyakov et al., 2012
pET15b IbpAΔN12	Overexpression of His-tagged *Al*IbpAΔN12 in *E.coli*	IbpA_N12 up- IbpA_C lw	This study
pET15b IbpAΔN25	Overexpression of His-tagged *Al*IbpAΔN25 in *E.coli*	IbpA_N25 up- IbpA_C lw	This study
pET15b IbpAΔN12C14	Overexpression of His-tagged *Al*IbpAΔN12C14 in *E.coli*	IbpA_N12 up- IbpA_C14 lw	This study
pET15b IbpAΔN25C14	Overexpression of His-tagged *Al*IbpAΔN25C14 in *E.coli*	IbpA_N25 up- IbpA_C14 lw	This study
pET15b IbpAΔC14	Overexpression of His-tagged *Al*IbpAΔC14 in *E.coli*	IbpA_N up- IbpA_C14 lw	This study
pET15b IbpAN11N12	Overexpression of His-tagged *Al*IbpAN11N12 in *E.coli*	IbpA_N11N12 up- IbpA_N11N12 lw	This study
pET15b IbpASEP	Overexpression of His-tagged *Al*IbpASEP in *E.coli*	IbpA_N up- IbpA_SEP lw	This study
pET15b IbpASEPΔN12	Overexpression of His-tagged *Al*IbpASEPΔN12 in *E.coli*	IbpA_N12 up- IbpA_SEP lw	This study
pET15b IbpASEPΔN25	Overexpression of His-tagged *Al*IbpASEPΔN25 in *E.coli*	IbpA_N25 up- IbpA_SEP lw	This study

Table S2. Primers used in this study

Primer	Sequence
IbpA_N up	5′ CTG GTG CCG CGC GGC AGC CAT ATG CTC GAG GAT CCG ATG TTG AGT TTA TTG AAC AAG AAT AGA AG 3′
IbpA_N12 up	5′ CTG GTG CCG CGC GGC AGC CAT ATG CTC GAG GAT CCG GAT GAT TTC TTC GAA GAC TTC AAT GTG C 3′
IbpA_N25 up	5′ CTG GTG CCG CGC GGC AGC CAT ATG CTC GAG GAT CCG ACT ACT TCT AAC TTA ATG AGA ACA G 3′
IbpA_C lw	5′ CCA ACT CAG CTT CCT TTC GGG CTT TGT TAG CAG CCG GAT CCT TAT TTA AGT TCT AAA TAA CGT TTT TCA GGC 3′
IbpA_C14 lw	5′ CCA ACT CAG CTT CCT TTC GGG CTT TGT TAG CAG CCG GAT CCT TAT TTT GGA AGT TCG ATA TGT AAC ATA CCG 3′
IbpA_N11N12 up	5′ CAA GAA TAG AAG TAA CAA TGA TGA TTT CTT CGA AGA C 3′
IbpA_N11N12 lw	5′ GTC TTC GAA GAA ATC ATC ATT GTT ACT TCT ATT CTT G 3′
IbpA_SEP lw	5′CCA ACT CAG CTT CCT TTC GGG CTT TGT TAG CAG CCG GAT CCT TAT TTG GGT TCG GAA TAA CGT TTT TCA GGC 3′

Table S3. The identity of full-length proteins and their features for *Al*IbpA compared to *Ec*IbpA or *Ec*IbpB

	Full-length	N-termini	ACD	C-termini
*Ec*IbpA	18.0%	18.0%	22.0%	29.4%
*Ec*IbpB	20.3%	20.3%	28.1%	12.5%

Table S4. The temperature stability (Tm_{50}) of various proteins

Protein	Can No (Sigmaaldrich)	Tm_{50}, °C
Alcohol dehydrogenase	55689	53±1.1
Bovine insulin	I6634	48±5.1
Human transferrin	T3705	58±2.3
Trypsin inhibitor	10109886001	73±1.2
Catalase	C9322	53±2.5
*Al*IbpA	-	53±4.5

Table S5. The chaperone-like activities (Tm_{50}) of full-length and truncated IbpA proteins

*Al*IbpA variant	Protein structure	Tm_{50}, °C	*Al*IbpA variant	Tm_{50}, °C
			Insulin	48±5.1
*Al*IbpA	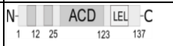	53±4.5	Insulin +*Al*IbpA	53±5.7
*Al*IbpAΔN12	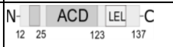	56±5.2	Insulin +*Al*IbpAΔN12	58±6.7
*Al*IbpAΔN25	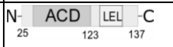	56±5.4	Insulin +*Al*IbpAΔN25	58±6.9
*Al*IbpAN11N12	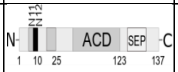	58±6.1	Insulin +*Al*IbpAN11N12	59±4.5
*Al*IbpAΔC14	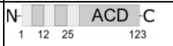	46±5.2	Insulin +*Al*IbpAΔC14	45±5.7
*Al*IbpAΔN12C14	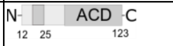	48±4.1	Insulin +*Al*IbpAΔN12C14	42±5.6
*Al*IbpAΔN25C14	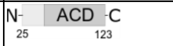	40±4.8	Insulin +*Al*IbpAΔN25C14	39±3.5
*Al*IbpASEP	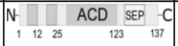	51±4.6	Insulin +*Al*IbpASEP	50±4.7
*Al*IbpASEPΔN12	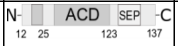	52±6.6	Insulin +*Al*IbpASEPΔN12	51±5.1
*Al*IbpASEPΔN25	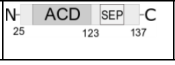	54±5.9	Insulin +*Al*IbpASEPΔN25	53±7.3

[1] Determined by measuring the SYPRO Orange fluorescence

Table S6. Distribution of various oligomeric fractions (%) in solutions of full-length *Al*IbpA and various truncated versions of the protein

		Distribution of various oligomeric fractions, % Determined from gel-filtration data			Distribution of various oligomeric fractions, % Determined by quantification of TEM-images	
*Al*IbpA variant	Protein structure	I peak	II peak	1×-4×-mers	I peak	II peak
*Al*IbpA	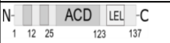	23±10.3	74±17.8	3±0.2	26±4.1	74±15.3
*Al*IbpAΔN12	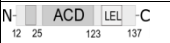	88±17.1	10±1.1	3±0.1	83±23.1	17±3.5
*Al*IbpAΔN25	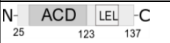	77±21.3	12±2.4	11±2.1	85±17.9	15±3.1
*Al*IbpAN11N12	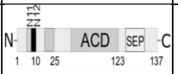	8±1.7	88±12.2	4±0.7	36±8.5	64±15.7
*Al*IbpAΔC14	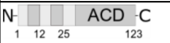	7±4.1	86±23.4	7±2.3	21±4.6	79±15.3
*Al*IbpAΔN12C14	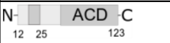	1±0.1	86±22.8	14±10.3	7±2.5	93±21.6
*Al*IbpAΔN25C14	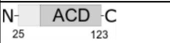	1±0.2	50±13.5	49±10.3	1±0.3	99±24.8
*Al*IbpASEP	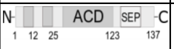	18±4.4	56±7.8	26±5.9	33±7.9	67±20.3
*Al*IbpASEPΔN12	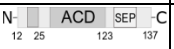	2±0.8	59±10.1	39±9.9	11±2.2	89±18.9
*Al*IbpASEPΔN25	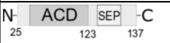	69±15.5	23±3.2	8±2.0	96±23.6	4±0.8

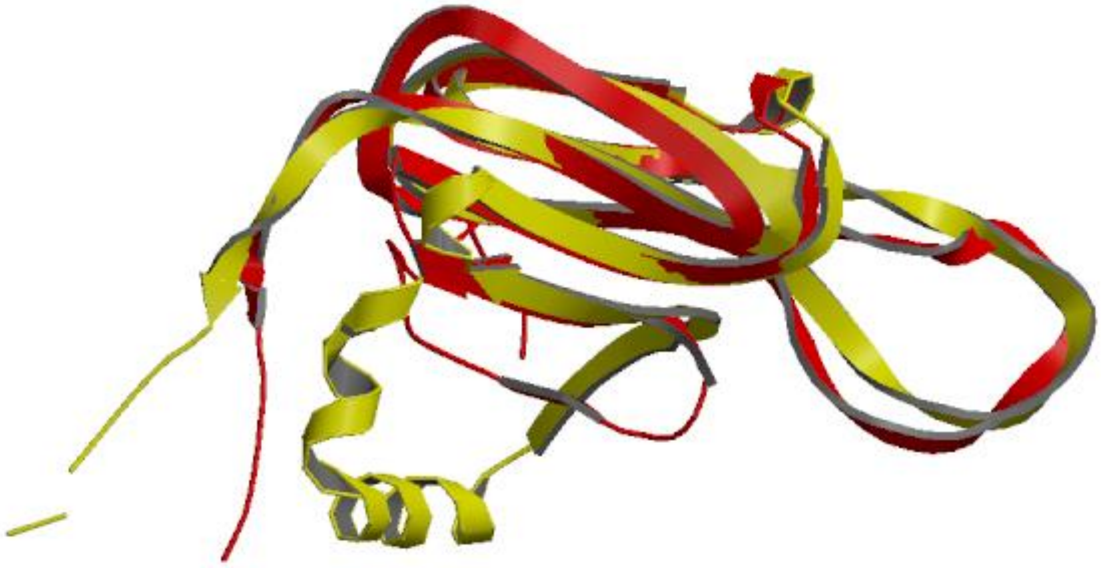

(a)

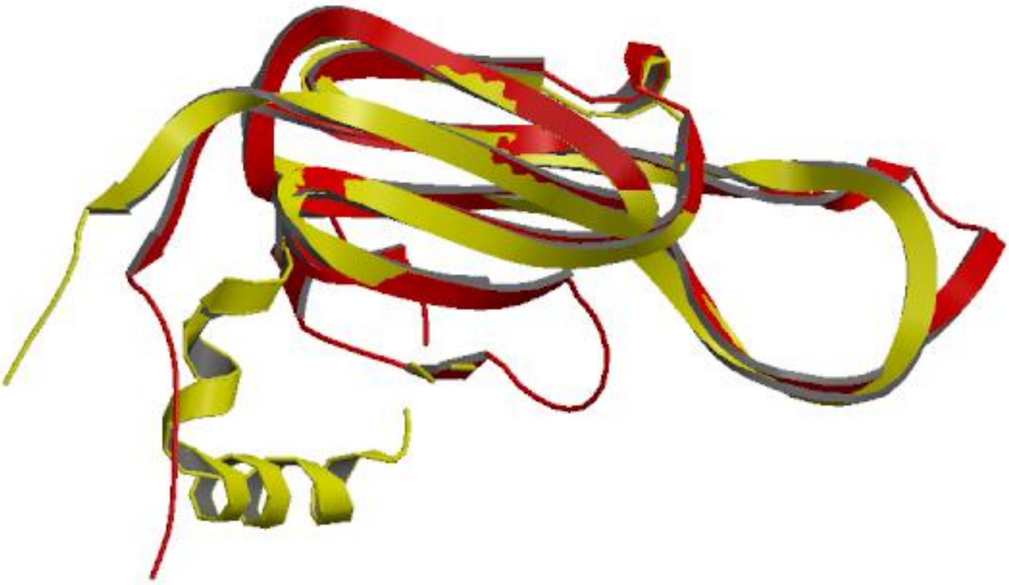

(b)

Figure S1. The models of tertiary structures of sHSPs from *A. laidlawii* (red) and *E.coli* (yellow) were obtained by using Phyre2 server and their superposition was obtained with SuperPose online software. (**a**) – superposition of *Al*IbpA and *Ec*IbpA, (**b**) – superposition of *Al*IbpA and *Ec*IbpB.

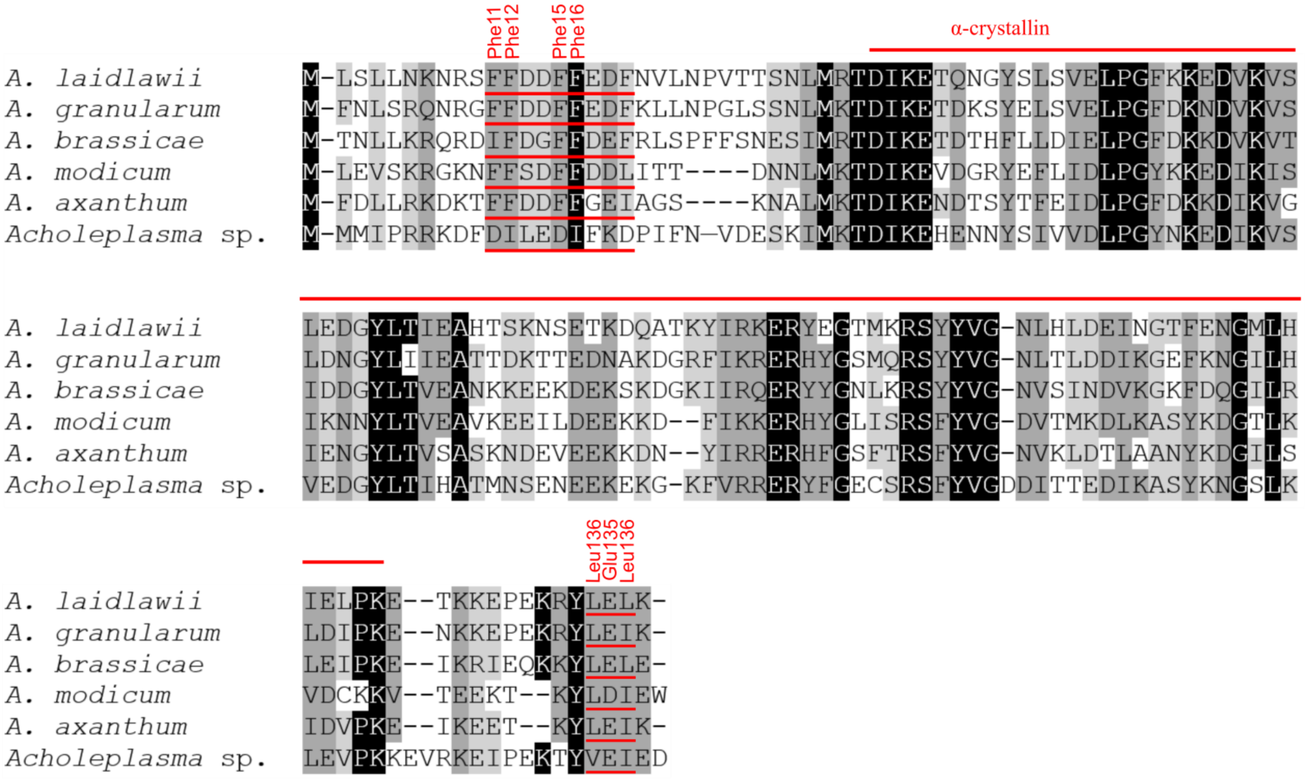

Figure S2. Multiple alignment of IbpA proteins from various *Acholeplasma spp.* Residues highlighted in gray show homologous substitutions. Amino acids in black represent similar residues. Putative (W/F)(D/F) PF and V/IXI/V motifs are underlined.

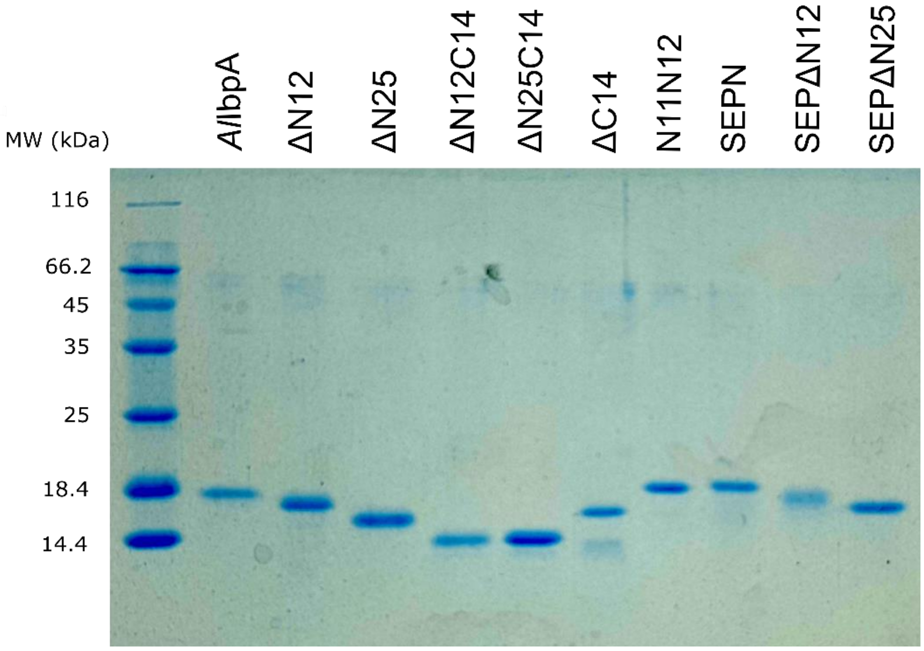

Figure S3. The SDS-PAGE analysis of purified recombinant truncated/mutated *Al*IbpA-His_6 proteins produced in *E.coli* BL21.

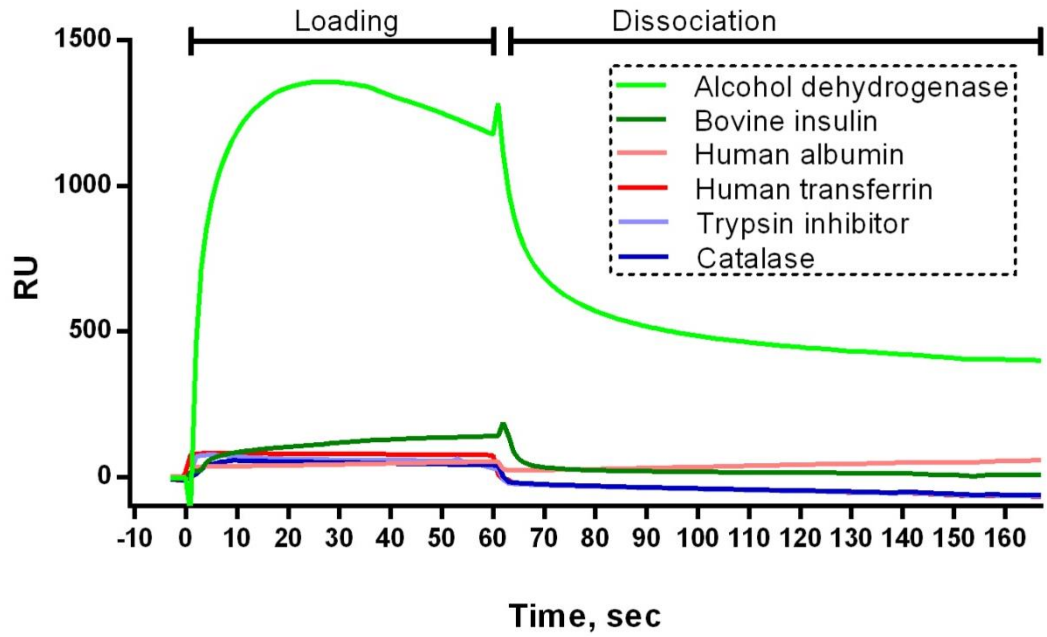

Figure S4. The SPR-analysis of various proteins interaction with *Al*IbpA. *Al*IbpA-His_6 was immobilized on NTA chip until ~2000 resonance units (RU). All proteins (1 mg/ml solution in running buffer) were injected in a volume of 15 μl with a flow rate of 15 μl/min.
